# Using causal loop diagrams to examine the interrelationships between factors influencing family planning utilisation in urban east central Uganda

**DOI:** 10.1136/bmjgh-2024-016342

**Published:** 2025-08-17

**Authors:** Jacquellyn Nambi Ssanyu, Rachel Cassidy, Leif Eriksson, Joan Kalyango, Peter Waiswa, Mats Målqvist

**Affiliations:** 1 Centre for Health and Sustainability, Women's and Children’s Health, Uppsala Universitet, Uppsala, Uppsala, Sweden; 2 Health Policy Planning and Management, Makerere University School of Health Sciences, Kampala, Kampala, Uganda; 3 KPM Center for Public Management, University of Bern, Bern, Switzerland; 4 Clinical Epidemiology Unit, Makerere University College of Health Sciences, Kampala, Kampala, Uganda; 5 School of Public Health, Makerere University, Kampala, Uganda

**Keywords:** Health systems, Maternal health, Public Health, Other study design, Global Health

## Abstract

**Introduction:**

Despite progress in reducing Uganda’s unmet need for family planning (FP), particularly in urban areas, it remains high with notable intraurban disparities. FP services in urban settings are delivered in a complex health system, which impacts service delivery and utilisation. Acknowledging the complexity of FP utilisation in these contexts, this study adopted a systems thinking approach, using causal loop diagrams (CLDs), to examine the interrelationships between the factors influencing FP uptake in urban east central Uganda.

**Methods:**

This qualitative study, conducted in Jinja city and Iganga municipality, used community-based system dynamic modelling to create CLDs to visualise the interrelationships between the different factors. The CLDs were developed through two group model building workshops, involving 14 community members and other key stakeholders. Initial model building was based on themes derived from analysis of data from eight focus group discussions, eight key informant interviews and four indepth interviews. The resulting CLDs were subsequently validated in a separate meeting with the participants.

**Results:**

The study identified 30 key factors influencing FP utilisation mediated through five mechanisms: reproductive autonomy, service access, client satisfaction, perceptions of FP as important and perceived susceptibility to sexually transmitted infections among women. It highlighted the role of self-regulating feedback loops related to side effects, commodity and supply availability and provider workload, which moderate FP use. Additionally, the study emphasised the positive reinforcing effects of enhanced access to FP information on service access and uptake.

**Conclusion:**

Effective FP intervention designs should account for the complex interplay of factors affecting utilisation. Key leverage points include addressing the underlying negative religious and sociocultural beliefs that shape system behaviour, improving information flow and data use for better commodity management and human resource sustainability, enhancing contraceptive pharmacovigilance systems, improving the management of side effects and increasing access to FP information.

**WHAT IS ALREADY KNOWN ON THIS TOPIC**

- Despite progress in reducing the unmet need for family planning (FP) in Uganda, it is still high and intraurban disparities persist.
- While the factors that influence FP utilisation are well known, a detailed understanding was lacking about how these interact within complex emergent urban health systems in Uganda to influence the use of FP.

**WHAT THIS STUDY ADDS**

- Using community-based system dynamics modelling, this study developed causal loop diagrams, revealing the key mechanisms through which the different factors influence FP use in urban settings.
- The study also highlighted the role of self-regulating feedback loops related to side effects, commodity and supply availability and provider workload, which moderated FP use and emphasised the positive reinforcing effects of enhanced access to FP information on service access and uptake.

**HOW THIS STUDY MIGHT AFFECT RESEARCH, PRACTICE OR POLICY**

- This study identified key leverage points to guide future design of FP interventions in urban low-resource settings.
- The study also advocates for integrated approaches that consider the multifaceted influences on FP utilisation for more effective FP programmes.

## Introduction

 The world’s urban population is projected to increase from 55% (in 2018) to 68% by 2050, with 90% of this growth happening in Asia and Africa.^1^ Uganda exemplifies this urbanisation trend, experiencing a 5.2% annual urbanisation rate, with half of its population expected to reside in urban areas by 2050.^2^ Despite the advantages urbanisation offers,^3^ its impact on poverty in Sub-Saharan Africa has been limited,^4^ evidenced by the proliferation of slums and informal settlements where 48% of Uganda’s urban population lives.^5^ Although rural-urban migration contributes to this trend, part of the urban population growth in low- and middle-income countries (LMICs) stems from natural population increase.^6^ Family planning (FP) could play a role in fostering economic growth in these urban areas by granting individuals control over birth timing and spacing.^7^ This not only safeguards reproductive rights but also prevents unintended pregnancies and reduces unsafe abortions, promoting women’s and children’s health, poverty reduction and environmental sustainability.^8^,^12^

Despite these benefits, 48% of global pregnancies are unintended.^13^ In Uganda, specifically, 28% are mistimed, 6% are unwanted and adolescent pregnancies stand at 24%.^14^ These statistics contribute to the 39 induced abortions per 1000 women annually, with abortion-related complications accounting for 7% of maternal deaths.^15 16^ While the unmet need for FP in Uganda has decreased from 35% in 2000 to 24% in 2022 (among married women),^14^ it remains higher than in neighbouring countries Kenya (18%), Rwanda (19%) and Tanzania (22%).^17^ Moreover, within-country disparities persist. Despite the comparatively higher modern contraceptive use in urban areas (42.8% vs 36.3% in rural areas) and lower unmet need for FP (19.4% vs 25.7%),^14^ intraurban disparities exist. Informal settlements within an urban municipality in central Uganda, for instance, reported an unmet need for FP of 37.3%,^18^ surpassing both the national average and that in rural areas.

Modern FP utilisation is influenced by several factors including sexual reproductive health policies; service aspects like provider-client interactions and contraceptive method choices; individual factors such as education and gender and social norms.^19^ In urban contexts, FP services are delivered in complex health systems with multiple interacting actors and processes influencing service uptake.^20^ Particularly, rapidly urbanising LMICs face plurality in providers with limited free primary health care.^21^ The urban health system is also embedded within a dynamic social system where institutions and stakeholders interact repeatedly over time, often within a resource-constrained environment.^22^ These interactions generate complex, dynamic, emergent behaviour, impacting service delivery and the effectiveness of FP interventions, possibly contributing to the intraurban inequalities.

While many of the factors influencing modern FP use in Uganda are well-documented,^23^,^28^ understanding the interrelationships between them, particularly in urban settings, remains limited, hindering targeted programme design and implementation. Adopting a systems thinking approach can assist in unravelling this complexity, to aid understanding of what works, for whom and under what circumstances.^29^ This approach views systems and their components as closely interrelated, emphasising the interpretation of interactions within and between systems.^30^ It goes beyond singular events or standalone factors, exploring behavioural patterns and the underlying systemic interrelationships responsible for these patterns.^30^

Systems thinking approaches like system dynamics modelling and tools like causal loop diagrams (CLDs) offer valuable insights into complex system interrelations. Their holistic systems approach facilitates a comprehensive understanding of impact pathways for system-strengthening programmes, identifying potential spillover effects and unintended consequences.^31^ CLDs, in particular, are visual tools that use variables, arrows and polarity symbols to map how different factors in a system influence one another. A plus sign (+) on an arrow in a CLD indicates that an increase in one variable leads to an increase in the next, while a minus sign (–) shows an inverse relationship. These connections often form feedback loops, which can be reinforcing (amplifying changes in the system) or balancing (stabilising the system by counteracting change). By making these dynamic relationships visible, CLDs help stakeholders gain clarity regarding their mental models about the factors influencing specific behaviours within a given system.^32^ Moreover, CLDs facilitate the identification of key leverage points within a system, the locations where minor interventions can yield significant effects.^32 33^

Several studies have used CLDs to analyse complex health system issues. For instance, Varghese *et al*^34^ used CLDs to illustrate the intricate nature of childhood immunisation coverage in India, while Baugh Littlejohns *et al*^35^ employed CLDs to analyse the influences on health promotion, policy and practice within Australia’s health system, revealing leverage points for improvement. Cassidy *et al*^36^ also applied CLDs in Tanzania to study how payment for performance impacts the delivery and uptake of maternal and child health services. In Uganda, specifically, CLDs were used to explain the factors contributing to the stagnation of neonatal mortality rates^37^ and why dual health worker practice persists.^38^ Despite these studies, the application of CLDs in understanding the dynamics of FP use, a multilayered issue that intersects with broader societal concerns like reproductive rights, health systems and socioeconomic development, has been limited.

In light of Uganda’s target to reduce the unmet need for FP to 15% by 2025,^39^ especially in the context of rapid urbanisation, it is crucial to ensure that interventions are not only targeting the right issues but are also responsive to the diverse needs of all subpopulations. This study, recognising the complexity of FP utilisation in urban contexts, adopted a systems thinking approach to examine the interrelationships between the factors that influence modern FP uptake in urban east central Uganda. The insights gained from this analysis were used to identify leverage points, which could inform the design and optimisation of FP interventions to enhance their effectiveness.

## Methods

### Setting

This study was performed in Jinja city and Iganga municipality as part of a larger project, the Urban Thrive Project,^40^ implementing a package of health system-strengthening interventions to increase the uptake of voluntary FP in urban eastern Uganda. Jinja and Iganga are located in the Busoga subregion of eastern Uganda. Busoga has an adolescent pregnancy rate of 20.7% and a total fertility rate of 5.7 children per woman, above the national average of 5.2.^14^ The subregion is also home to the majority of poor persons in the country and one of the top contributors to total national poverty (14.1%).^41^ Jinja city, located on Lake Victoria’s northern shores and along the River Nile, is a commercial hub and tourist destination that was designated as a city in 2021. It is Uganda’s second-largest city with a population of 83 400 people, including a substantial migrant community.^42^ Iganga is a neighbouring growing town in Iganga district, with a population of about 65 500 people.^42^ This work was done as part of an ongoing collaboration and prioritised equitable collaboration with LMIC researchers, as highlighted in the reflexivity statement (online supplemental Table S1).

### Study design

Using a qualitative design, this study applied community-based system dynamics (CBSD) modelling to investigate the interrelationships between the factors influencing FP uptake. CBSD was used to develop CLDs to visually represent the interconnections and feedback loops shaping these factors. As a participatory method, CBSD engages communities in the process of understanding and changing systems from the feedback perspective of system dynamics with a focus on analysing and solving problems that involve dynamic complexity.^32^

### Data collection and analysis

Hovmand’s^32^ three-stage approach to CBSD modelling—consisting of: (1) problem scoping and identification, (2) core modelling team (CMT) planning and capacity building and (3) group model building (GMB) workshops—was adopted. This process followed preprepared scripts adapted from Scriptapedia^43^ and Ager *et al*^44^ to standardise the data collection process, ensuring consistency, efficiency and quality across the different tasks.

### Problem scoping and identification

An analysis of qualitative data collected during the Urban Thrive Project’s baseline assessment (November 2021 to January 2022) was done to examine the factors influencing FP utilisation in Jinja and Iganga. The data comprised 20 key informant interviews (KIIs), 24 in-depth interviews (IDIs) and 17 focus group discussions (FGDs) transcripts. The IDIs and FGDs involved men, women and adolescents (15–19 years old) across the two locations. Separate FGD sessions were conducted for each group to ensure targeted discussions. The KIIs included reproductive health service providers and city/town leaders. Further details on study participants and procedures are available in the project protocol.^40^

For this study, FGD and interview transcripts were purposively sampled for analysis from the Urban Thrive Project dataset to give a representation of participants across age groups, genders and sites. Efforts were made to include FGDs and interviews involving vulnerable and key populations such as slum residents and island/fishing communities, as well as commercial sex workers. The lead investigator reviewed the transcripts for accuracy, comparing them to the original audio recordings, and later conducted reflexive thematic analysis^45^ using QualCoder V.3.3 software for data processing. First, the transcripts were read repeatedly for data familiarisation. Subsequently, codes were assigned to relevant segments of the data and grouped based on similarities and patterns to identify potential themes. The themes were then refined to ensure they accurately reflected the data. They were later defined and supported by evidence from the data. The analysis included eight FGD, eight KII and four IDI transcripts (online supplemental Table S2). These were selected based on thematic saturation, the point at which no new themes were emerging from the data. The themes generated provided an initial structure for model development.

### CMT planning and capacity building

A CMT of six, two men and four women, was established to oversee the design of the GMB workshops. Team members were selected, working with community health workers in the target communities, for their diverse expertise, process tolerance and ability to commit time to the study. They were also selected based on their knowledge of FP service delivery, understanding of community needs, ability to navigate cultural dynamics and capacity to ensure culturally appropriate and feasible activities. The team included two Urban Thrive Project staff who were familiar with project objectives, a community member for local perspectives, two FP service providers and the lead investigator who guided the research process. The CMT held three meetings to familiarise themselves with systems thinking and plan the GMB workshops. In the first meeting, the lead investigator introduced the project, explained systems thinking principles and presented the themes from qualitative data analysis. These themes were refined into measurable variables to allow conversion of abstract concepts into quantifiable elements that could easily be represented in the CLDs. The second meeting focused on identifying stakeholder groups to invite to the GMB workshops and finalising the workshop process map. During the third meeting, the CMT developed detailed workshop agendas, specifying activities, timings and participant roles. This agenda was used to finalise the facilitation manual containing project descriptions, agendas, team roles and scripts. CMT roles were assigned, including modeller facilitator, community facilitator, process coach, note-taker and debriefer, with a rehearsal session conducted to refine scripts and ensure smooth facilitation.

### GMB workshops

Two full-day workshops were held a week apart. These involved several exercises, both convergent, aimed at finding common ground and divergent, focused on exploring diverse perspectives, along with facilitated discussions. The workshops included 14 participants selected to ensure gender and age representation from Jinja and Iganga residents aged 15 years and older (table 1). Participants were selected based on their experience with accessing and using FP services in the study areas. Specifically, individuals were chosen if they had either direct experience with FP services or had actively interacted with service users. Community health workers assisted in identifying these participants. The same participants were engaged in both workshops. Discussions were held in English and the local languages commonly spoken in the area, Lusoga and Luganda, based on feedback from the participants. Key conversations were recorded by the note-taker, while all workshop proceedings were audio recorded.

**Table 1:** GMB workshop participant characteristics

Characteristic		Frequency (n=14)
Site		
	Iganga municipality	6
	Jinja city	8
Age		
	16–24 years	3
	25–34 years	3
	35–44 years	5
	45–67 years	3
Gender		
	Female	10
	Male	4
Marital status		
	Married/cohabiting	8
	Unmarried/widowed	6
Religion		
	Anglican	6
	Catholic	2
	Muslim	1
	Pentecostal	5
Education level		
	Secondary school	3
	Certificate level	4
	Diploma level	3
	Degree and higher	4
Participant category		
	Community member	5
	Community health worker	2
	District FP focal person	1
	Health worker	3
	Urban Thrive Project staff	2
	School teacher	1
Residence		
	Slum residence	2
	Non-slum residence	12

FP, family planning; GMB, group model building.

#### Workshop 1

In the first workshop, participants were introduced to the project and familiarised with complex systems and systems thinking using the ‘blind men and elephant parable’, in the Elephant Script.^46^ The exercise proved highly effective in explaining systems thinking and its relevance. Divided into Jinja and Iganga groups, participants then created ‘rich pictures’ depicting all elements, relationships and interactions related to FP in their respective communities, which were then discussed in a plenary session. The rich picture from the Jinja group is presented as online supplemental figure S3. Notably, the rich pictures and discussions from both groups revealed similar issues. The community facilitator then presented the variables from the qualitative analysis, illustrating them with data quotes and seeking validation from the participants. The group was encouraged to identify additional factors, primarily from the rich pictures.

Variable names and definitions were further clarified and refined. The variables were written onto sticky notes on a board, and each participant received 12 dot stickers to mark the variables they deemed most important for improving FP uptake in their community. Variables with no or minimal votes underwent discussion within the larger group. If unanimously agreed on as less relevant within their communities or outside the boundary of the project, these variables were removed. Additionally, variables addressing similar elements were merged for coherence.

To encourage deeper thinking about FP dynamics, participants were split into two groups to develop graphs-over-time reflecting changes in select variables’ behaviour. The x-axis was marked as ‘time’, considering changes over the past 10 years and projecting expected behaviour by 2025 assuming all other factors remained constant, alongside desired behaviour. On the y-axis, variable names were scaled from ‘low’ to ‘moderate’ to ‘high’, which made the concepts more relatable and comprehensible. Each group presented its graphs, summarising the problem’s dynamics from its perspective. The modeller facilitator then presented a reference mode depicting past modern contraceptive use, based on Uganda Demographic and Health Survey data, and anticipated behaviour by 2025.

All variables that were prioritised as important from the previous exercises were written on sticky notes and placed in a circle. Participants discussed relationships between variables, drawing directional arrows denoting influences. When bidirectional relationships arose, the dominant influence was considered. This process created an interrelationship diagraph (IRD), a useful precursor to drawing CLDs. The IRD drawn from the workshop is provided as online supplemental figure S4.

#### Workshop 2

All 14 participants involved in the first workshop took part in the second. The workshop started with a refresher on CLDs and their interpretation, ensuring readiness for causal mapping. CLDs have two components: variables and influences (links). Influences are indicated by arrows showing the direction of influence and polarities (plus or minus signs), which show how one variable influences another. Plus signs indicate that an increase in the cause variable leads to an increase in the effect variable, assuming other factors remain constant, while minus signs indicate a decrease. Feedback loops occur when arrows connect a variable to itself through a series of other variables and can be either balancing (labelled as B) or reinforcing (labelled as R). Balancing loops arise where there is an attempt to resolve a problem, while reinforcing loops show a growing action where each action adds to another.^32 37^ Reinforcing loops can result in either virtuous or vicious cycles: virtuous cycles indicate a continuous growth pattern, where positive changes in one variable lead to further desirable changes, whereas vicious cycles represent a downward spiral, where negative changes in one variable lead to further negative changes. Delays in CLDs are denoted by arrows with equal signs, indicating temporal gaps between cause and effect.

Using two main outcomes and one key driver from the IRD, a seed model was created as a foundational structure for developing the CLD. The seed model was presented on wall charts, encouraging participants to reflect on variable interactions in their communities. Each participant was encouraged to identify cause-and-effect relationships between variables and the seed model’s key factors. Sticky notes with variables were added to the charts, linked with arrows indicating influence direction and polarity.

While some participants initially expressed concerns about their ability to contribute fully to the workshop due to the perceived complexity of the process, the use of relatable tools like the ‘blind men and elephant’ parable and interactive exercises such as rich pictures and graphs-over-time helped them navigate through most exercises with ease. The sessions were also carefully structured, with facilitators providing ongoing support at every stage. The community facilitator played a key role in ensuring instructions were understood, addressing questions in real time and clarifying concepts as needed. Whenever participants struggled with certain connections, facilitators paused discussions to re-examine relationships or revisit key variables, reinforcing understanding before moving forward. Additionally, the process coach monitored engagement and intervened when confusion arose. Through this iterative approach, participants steadily gained confidence, shifting from initial uncertainty to active engagement.

### CLD refinement

The CLD drawn on wall charts during the workshop (online supplemental figure S5) was transferred to Vensim Personal Learning Edition V.9.3.5 for refinement. 33 variables were selected for inclusion into the CLD during the GMB exercises. This CLD, presented in online supplemental figure S6), was further reviewed and refined by the CMT and research team, incorporating additional insights from the GMB recordings. Evaluating the diagram’s coherence, each cause-and-effect relationship was analysed, ensuring a logical narrative. This assessment identified balancing and reinforcing loops. Variables that influence the system without being influenced by others, indicated by the absence of incoming causal arrows in the CLD, were considered exogenous drivers. In addition, hubs were identified as variables with the highest number of causal links.

### CLD validation

Participants who took part in the workshops were invited to a validation meeting 3 weeks later. However, four did not participate in the session due to other commitments. In this meeting, they were presented with the refined CLD for review. They engaged in discussions to determine whether the model aligned with previous discussions. Based on their feedback, the CLD was refined further by adjusting variable connections and revising the direction or polarity of certain relationships to better reflect their perspectives.

### Techniques to enhance trustworthiness

The use of CBSD modelling allowed for deep engagement with stakeholders, ensuring that diverse perspectives were considered and that the model development was rooted in real-world contexts. The iterative nature of the workshops and the follow-up validation meeting helped to refine and confirm the model developed, ensuring that it accurately represented the community’s views and experiences, enhancing credibility of the study. Additionally, the application of standardised scripts during data collection enhanced the dependability of the process by ensuring consistency and reproducibility.

## Results

### Model description

**Figure 1:**
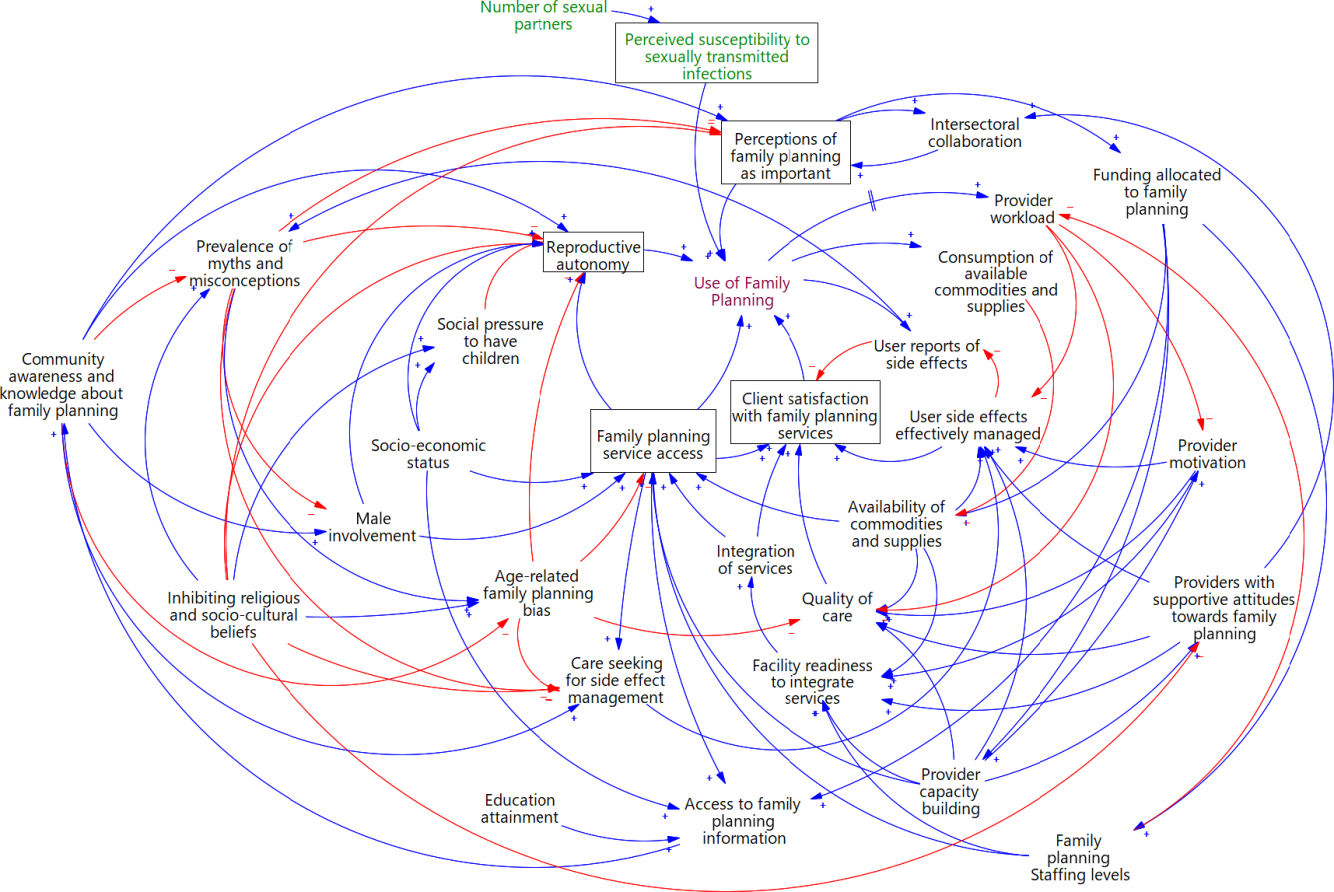
A causal loop diagram showing the interrelationships between the factors that influence family planning use in Jinja and Iganga. Variables in green are those that apply to women and not men. Variables shown in boxes represent the key pathways through which the different factors influence family planning use. Red arrows indicate links with negative polarities while those in blue represent links with positive polarities.

The final CLD includes 30 variables (figure 1), illustrating both enabling and inhibiting factors that influence FP use. These factors operate through five key drivers or mechanisms (detailed in subsequent sections): (1) reproductive autonomy, (2) FP service access, encompassing affordability (alignment of service costs with clients’ means), accessibility (ease of reaching services), acceptability (how comfortable clients are with the characteristics of the provider and vice versa) and accommodation (service organisation to meet client needs); (3) client satisfaction with services; (4) perceptions of FP as important and (5) perceived susceptibility to sexually transmitted infections (STIs) (among women). These central nodes are highly interconnected and feature in the main feedback loops that drive system behaviour. Meanwhile, socioeconomic status, education attainment, number of sexual partners and inhibiting religious and sociocultural beliefs were identified as exogenous drivers which defined the system’s boundaries.

### Reproductive autonomy

Reproductive autonomy, the ability of individuals to make independent and informed decisions about using FP, was identified as a key factor driving its uptake (figure 1). Increased knowledge about FP and male involvement reinforced this autonomy. Additionally, increased access to a range of FP methods and services was said to empower individuals to exercise greater control over their reproductive choices. Conversely, social pressure to have children reduced reproductive autonomy. This pressure intensified with higher socioeconomic status and stronger religious and sociocultural beliefs that oppose FP. Furthermore, age-related biases, mainly related to young people and older women’s use of FP and myths and misconceptions, further limited reproductive autonomy.

### FP service access

FP use was also shown to be directly influenced by service access, which was positively affected by several factors. These included increased availability of FP commodities and supplies, male involvement, a higher socioeconomic status, increased provider capacity building, integration of FP with other health services and an increase in FP staffing levels. In contrast, biases among health workers in deciding who should access FP based on age restricted access for young people and older women, the demographics reported to be affected by age-related bias.

### Client satisfaction

Client satisfaction also emerged as a crucial determinant of FP use. Increased access to FP services, integration of FP services, increased quality of care and effective management of contraceptive-related side effects increased satisfaction, driving higher use. On the contrary, increasing experiences and reports of side effects reduced satisfaction, discouraging initiation and continued use.

### Perceptions of FP as important

Participants reported that heightened perceptions of FP as important contributed to an increase in its use. Negative perceptions were fuelled by prevalent myths and misconceptions and inhibiting religious and sociocultural beliefs. However, enhanced community awareness and knowledge regarding FP helped to counter these barriers. Strengthened collaboration between FP programmes and other sectors like education and planning was noted to increase perceptions of FP as important, which further fostered intersectoral collaboration. Furthermore, increased perceptions of FP as important, particularly among decision-makers, led to increased funding allocation for FP activities.

### Perceived susceptibility to STIs

Women, particularly in slums and fishing communities, disclosed engaging in non-monogamous relationships and extramarital affairs, often driven by financial considerations. In such scenarios, they emphasised the importance of using condoms to prevent not only pregnancy but also STIs. They perceived themselves at heightened risk of contracting such infections and were determined not to carry them into their main relationships. Men also acknowledged participating in non-monogamous relations, but condom use was not consistently reported as obligatory in such relationships.

### Key feedback loops

Several feedback loops were identified, with the key ones revolving around: (1) FP-related side effects, (2) availability of commodities and supplies, (3) access to FP information and (4) service provider workload.

### Side effects related to FP use

**Figure 2:**
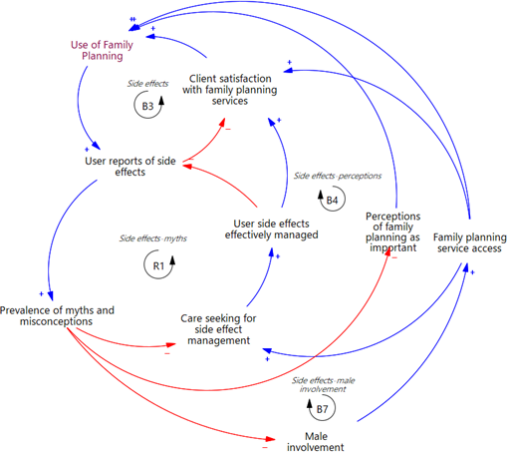
An extract from the causal loop diagram in figure 1 showing feedback loops related to the side effects of family planning use. Each balancing loop is denoted as *B#* (such as B3 and B4), and each reinforcing loop is labelled as *R# *(like R1). Arrows around the loop labels indicate the direction in which the loops should be read.

Given the inherent side effects of most modern FP methods, an increased user base results in a higher number of individuals experiencing and reporting side effects (balancing loop B3, in figure 2). Failure to address these side effects leads to user dissatisfaction, causing a decline in motivation to continue using FP. A similar outcome is observed in balancing loops B4 and B7 (figure 2), where increased user reports of side effects contribute to the spread of myths and misconceptions, leading to diminished community perceptions of its importance (B4) and reduced male involvement (B7). Diminished perceptions of FP’s importance lessen the number of people opting to use it, while decreased male involvement results in reduced support for partners’ FP use decisions, which ultimately restricts women’s access to FP services.

On the other hand, a vicious cycle of increased user reports of side effects is perpetuated through reinforcing loop, R1 (figure 2). When reports of side effects increase, there is a proliferation of myths and misconceptions. This, in turn, reduces care seeking to manage the side effects, negatively impacting effective management of side effects. Without adequate management, many side effects are unresolved, resulting in a continued increase in user reports of side effects and spread of myths and misconceptions.

### Commodity and supplies availability

**Figure 3:**
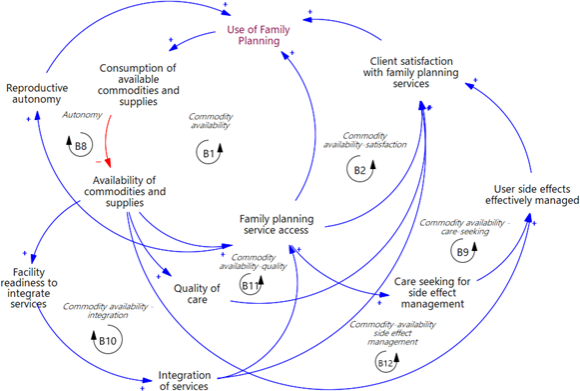
An extract from the causal loop diagram in figure 1 showing the feedback loops linked to commodity and supply availability.

Balancing loop B1 in figure 3 illustrates how commodity availability affects FP uptake. As FP use increases, the consumption of available commodities and supplies rises, leading to a decrease in their availability. This reduction hinders access to FP services, resulting in lower utilisation. Subsequently, the decrease in utilisation eases the consumption pressure, improving commodity and supplies availability and restoring the system to its initial state.

Similarly, loops B2 and B8 (figure 3) prevent increases in FP use through reduced client satisfaction and reproductive autonomy, respectively, with reduced access to services. As the consumption of available commodities and supplies increases, this reduces the availability of resources, especially supplies, to sustain quality care (B11) and effective side effect management (B12) (figure 3). This shortage then leads to decreased client satisfaction, ultimately reducing FP utilisation and returning it to its original level. Reduced satisfaction is also experienced through a decrease in service access due to reduced availability of commodities, which lowers care seeking for side effect management and eventual effective side effect management (B9). A parallel pattern is observed in balancing loop B10, where reduced commodity and supplies availability diminishes facility readiness for service integration, leading to decreased integration of FP services, subsequently impacting access and use of FP services.

### Access to FP information

**Figure 4:**
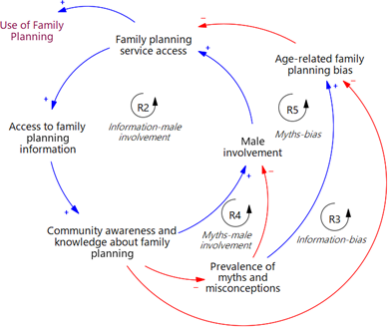
An extract from figure 1 highlighting key feedback loops related to access to family planning information.

An increase in access to FP information initiates a ripple effect: an increase in community awareness and knowledge about FP, mitigating age-related FP bias. This, in turn, opens up greater access to FP services for both young people and older women, providing them with increased avenues to obtain information about FP. This reinforcing feedback loop produces a virtuous cycle, enhancing access to services and subsequent increase in FP use (R3, in figure 4).

Enhanced access to information leads to increased knowledge and awareness, which in turn promotes greater male involvement when men are well-informed (R2). This positive feedback loop creates a virtuous cycle, amplifying access to FP services and information for both men and women. Additionally, increased awareness and knowledge reduce the prevalence of myths and misconceptions. This effect leads to an increase in male involvement (R4) and a reduction in age-related FP bias (R5, in figure 4). Both outcomes amplify access to services, creating a positive feedback loop that further intensifies access to information, increasing knowledge and awareness.

### FP service provider workload

**Figure 5:**
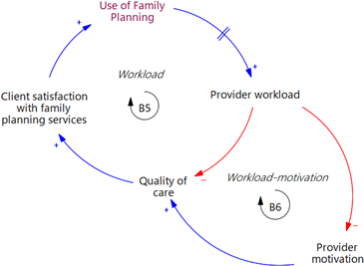
An extract from the causal loop diagram in figure 1 illustrating key feedback loops linked to family planning service provider workload.

Increase in the number of FP users over time heightens service provider workload, reducing the quality of care and subsequently lowering client satisfaction with the services provided, resulting in reduced FP use (B5, in figure 5). A comparable outcome occurs through a decline in provider motivation, also leading to a reduction in the quality of care (B6, in figure 5).

### Insights from participant feedback

Participants revealed that the GMB workshops improved their understanding of FP and the interconnectedness of the different factors. Community members appreciated the opportunity to ask questions and receive responses from the health workers present. A health worker from a facility not providing FP due to religious reasons expressed newfound appreciation for its benefits. She committed to educating clients about FP and referring interested individuals to facilities offering the services.

## Discussion

This study developed CLDs to visualise the interrelationships between factors influencing FP uptake in urban east central Uganda. 30 key factors were identified, operating through five primary mechanisms: changes in reproductive autonomy, FP service access, client satisfaction with services, perceptions of FP as important and perceived susceptibility to STIs among women. Furthermore, the study uncovered several feedback loops driving system behaviour, notably those pertaining to FP side effects, commodity and supplies availability, access to information and service provider workload.

The factors identified in this study align with prior research conducted in Uganda^2647^,^52^ and elsewhere.^53^,^56^ Notably, exogenous factors, socioeconomic status and religious and sociocultural beliefs influence several other factors more proximal to FP use. Their effects on FP uptake have also been documented in other studies.^57^,^60^ A surprising finding was the differential influence of non-monogamous relationships on FP use among men and women. Women in such relationships perceived higher susceptibility to STIs, driving the adoption of barrier methods. This finding is supported by earlier research in Eastern and Southern Africa, where women’s perceptions of HIV risk were shown to significantly influence condom use, while men’s perceptions had no such effect.^61^ Similarly, research from the USA found that women with multiple concurrent sexual partners were more likely to use contraception, particularly condoms, during periods of concurrency.^62 63^ This underscores the importance of integrating FP and STI prevention services, particularly in urban areas, amidst the HIV/AIDS threat. Such integration would not only benefit women but also men, who in most cases wield control over the timing and circumstances of sexual encounters and contraceptive use.^64^

Feedback loops linked to FP side effects were shown to play a pivotal role in the dynamics of FP use, primarily acting as self-corrective mechanisms to curb increases in FP users. Moreover, one reinforcing loop (R1, figure 2) perpetuates a vicious cycle, further amplifying user reports of side effects. Similar to our study, concerns about side effects have been cited as a barrier to FP use in Uganda^47^,^49^ and other countries.^53 55^ These concerns not only deter initial uptake but also restrict method choice,^65^ cause method switching and discontinuation,^66^ and reduce male partner support for contraceptive use, particularly when side effects affect sexual activity.^47^ Poor service quality during side effect management further exacerbates their impact on utilisation, such as refusals or delays to remove methods thought to cause the effects.^67 68^ Addressing side effects is crucial to optimising user satisfaction.

The availability of commodities and supplies was identified as a critical factor influencing FP utilisation through various self-regulating feedback loops. These loops prevent increases in FP use when commodity availability does not meet demand. They illustrate that, even with demand generation interventions, such as efforts to increase perceptions of FP as important by increasing knowledge and awareness, the number of users will naturally adjust to match the available commodities. Thus, low uptake of FP or limited utilisation of specific methods may not necessarily indicate low demand but could be a reflection of constraints in supplies and commodities. This influence of commodity security on FP use is also documented in Grindlay *et al’s*^69^ work in rural and urban settings in eastern and western Uganda. In contexts like Uganda, where over half of FP services are accessed through the public health sector^70^ via a centralised distribution system, these feedback loops represent a crucial leverage point for enhancing FP utilisation, particularly given the high prevalence of FP commodity stock-outs in Uganda.^69 71^

Research has shown access to FP information to increase modern contraceptive use,^72^ consistent with our findings. Our study revealed that enhanced access to information improves knowledge, which in turn promotes reproductive autonomy, emphasises FP’s importance, improves side effect management and increases access to services, which all increase FP use. The feedback loops in figure 4 highlight the reinforcing effects of information access and support its integration into FP programmes. Our findings also align with Dwomoh *et al*,^73^ who found that exposure to FP messages through traditional and electronic media was positively associated with contraceptive use across Sub-Saharan Africa. They recommend intensifying messaging through traditional channels while leveraging electronic platforms to encourage FP use. In urban Uganda, where mobile and social media use is expanding, such platforms offer promising opportunities to extend reach, counter misinformation and promote positive FP norms. These digital approaches, now recognised as promising high-impact practices in FP,^74^ can complement more conventional strategies by delivering timely, targeted content to diverse audiences. However, it is crucial to recognise the interplay of other factors influencing FP utilisation beyond access to information, as our study indicates. For instance, a study in informal urban settlements in Central Uganda found a high unmet need for FP (37.3%) despite widespread knowledge about FP,^18 75^ emphasising the need for multifaceted interventions that address barriers beyond merely information access.

The feedback loops related to workload underscore the challenge of meeting increasing demand while maintaining service quality. As user numbers grow, service providers face increased workloads, which may compromise care quality. Moreover, heavy client loads affect motivation, with potential for service quality erosion, which negatively affects client satisfaction and ultimately reduces FP utilisation. And, similar to our findings, other studies indicate that increased client loads degrade service quality.^76^,^78^ However, a study in rural Senegal found no such decline, though it did not account for other potential duties like administrative tasks that health workers might be involved in.^79^

Our findings can also be interpreted through the lens of the socio-ecological model, which is foundational to social and behaviour change programming.^80^ At the individual level, factors such as reproductive autonomy and knowledge influence FP decision-making. On the interpersonal level, male involvement was identified as an enabler. However, age-related bias and commodity availability highlight influences at the community and institutional levels. While policy factors were outside the scope of this causal mapping, their influence on outcomes observed at the institutional level, such as commodity availability and staffing levels, cannot be overlooked. The provider behaviour ecosystem map also provides valuable insights into the complexity of provider behaviour change in the FP context, highlighting the diverse factors that influence behaviour at the facility level and how they interact with one another.^81^ Similar to our findings, these frameworks demonstrate that changes at a single level may be insufficient for sustained FP uptake without addressing institutional, structural and provider-related barriers, reflecting the complex interplay of factors influencing behaviour at multiple levels.

### Key leverage points

Meadows^33^ provides guidance on how to identify and prioritise leverage points in complex systems to effect change. Applying this framework to the FP system in urban east central Uganda reveals several key leverage points. These points are presented below based on their potential influence as described in Meadows’ framework,^33^ starting with those likely to have the greatest systemic impact.

1. Changing paradigms: the most impactful leverage point involves shifting underlying paradigms, particularly addressing inhibiting religious and sociocultural beliefs. While changing beliefs can be challenging, Meadows^33^ recommends highlighting failures in old paradigms and putting change agents in visible positions of influence to facilitate this transformation.
2. Changing information flow structures: (a) analysing the commodity availability and provider workload loops indicates that improving FP information flow and data visibility at facility and national levels is a low-hanging fruit that could significantly improve FP use. This would improve monitoring and signalling for improved supply management and workload sustainability; (b) additionally, improved contraceptive pharmacovigilance would guide resource allocation for side effect management and inform efforts to develop safer contraceptives.
3. Addressing/leveraging reinforcing loops: (a) strategies to enhance information access significantly increase service access and utilisation; (b) furthermore, ensuring effective side effect management through interventions to encourage client care-seeking and provider capacity building for side effect management can reduce the gain of the negative ‘side effects-myths loop’, R1, in figure 2.

### Strengths and limitations

This study exhibits several strengths that enhance its relevance. Using CBSD modelling, the study incorporated stakeholders’ perspectives for a comprehensive system understanding. The GMB process positively impacted participants, fostering engagement and commitment, evidenced by the inquiries and feedback, including one participant’s pledge to start providing FP education. Furthermore, while the commonly used qualitative and quantitative research approaches effectively identify factors influencing FP use, many of which were also captured in the qualitative data analysis conducted as part of this study, CLDs facilitated a richer interpretation. Specifically, they highlighted the interrelationships between factors, revealing feedback loops that demonstrate how changes in one variable can ripple through the system and influence others over time. This systems thinking approach enabled a holistic understanding of the dynamics at play, helping to identify key leverage points where resources could be most effectively allocated to increase service uptake.

However, having only a single coauthor code the FGD and interview transcripts could have introduced unconscious bias into the interpretation of findings. Nonetheless, we believe this was mitigated by presenting the themes identified through data analysis to workshop participants for validation and refinement. This collaborative analysis enhanced the credibility of the analysis by ensuring that the identified themes resonated with participant perspectives and accurately reflected the data. Furthermore, while CLDs provide valuable visual representations of feedback loops and causal relationships, they are unable to quantify relationship strength. To address these limitations and enhance confirmability of the findings, a follow-on study is planned to develop a system dynamics model that will integrate numerical data, quantify feedback loops and enable simulation of dynamic behaviour to predict future outcomes under different scenarios. Additional research is also recommended to evaluate the effectiveness of interventions at the recommended leverage points in improving FP uptake.

## Conclusion

This study identified 30 key factors influencing modern FP utilisation in urban east central Uganda, mediated through various mechanisms. It highlights the role of self-regulating feedback loops related to side effects, commodity and supply availability and provider workload, which moderate increases in FP use. The study also emphasises the positive reinforcing effects of enhanced access to FP information on service access and uptake.

To increase FP uptake, the study identified several key leverage points, including addressing negative religious and sociocultural beliefs; improving information flow to inform commodity and supplies management and ensure human resource sustainability; enhancing pharmacovigilance systems for safe contraceptive use; improving community access to FP information and implementing effective side effect management strategies.

Ultimately, this study highlights the need to understand and address the complex dynamics that shape FP utilisation. By targeting these key leverage points, interventions can more effectively enhance access to and uptake of FP services, ultimately improving reproductive health outcomes in urban Uganda and similar settings globally.

## Supplementary material

10.1136/bmjgh-2024-016342 online supplemental file 1

10.1136/bmjgh-2024-016342 online supplemental file 2

10.1136/bmjgh-2024-016342 online supplemental file 3

10.1136/bmjgh-2024-016342 online supplemental file 4

10.1136/bmjgh-2024-016342 online supplemental file 5

10.1136/bmjgh-2024-016342 online supplemental file 6

## Data Availability

Data are available upon reasonable request.
