# Supplementary material for: Using causal loop diagrams to examine the interrelationships between factors influencing family planning utilisation in urban east central Uganda
Source: BMJ Glob Health. 2025 Aug 17;10(8):e016342. doi: 10.1136/bmjgh-2024-016342 (PMC12359470; doi:10.1136/bmjgh-2024-016342)

Supplemental Figure S3: A Rich Picture drawn by the Jinja city group

The image depicted household dynamics concerning couple communication and decision-making, the involvement of Community Health Workers or Village Health Teams (VHTs) in community education, obstacles encountered in accessing healthcare facilities, challenges in information and service accessibility among adolescents (especially those in school), the burden of high client volume and long waiting times at facilities, and the consequences of unintended pregnancies.

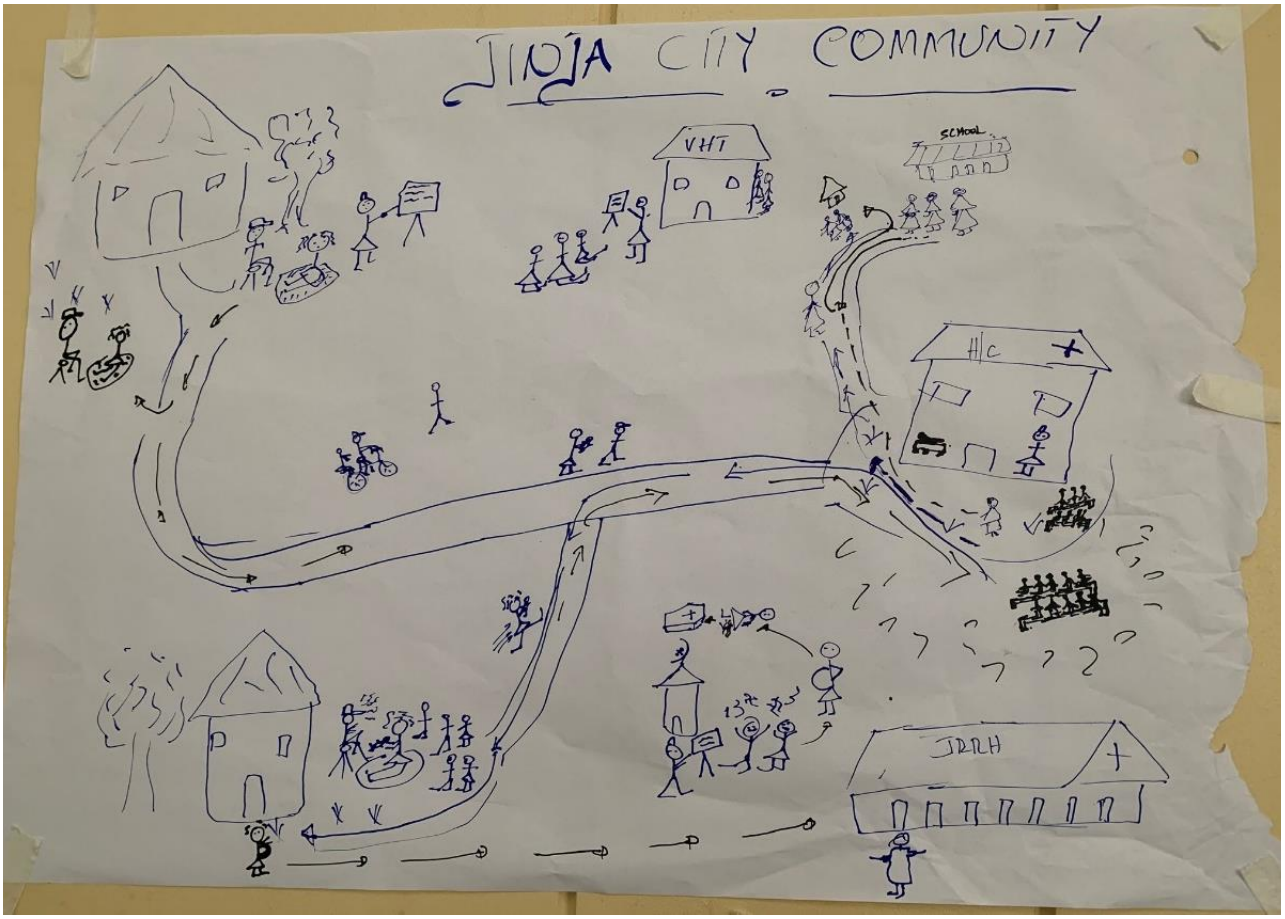

Supplement: online supplemental file 1 [file bmjgh-10-8-s001.pdf]
