# Supplementary material for: Using causal loop diagrams to examine the interrelationships between factors influencing family planning utilisation in urban east central Uganda
Source: BMJ Glob Health. 2025 Aug 17;10(8):e016342. doi: 10.1136/bmjgh-2024-016342 (PMC12359470; doi:10.1136/bmjgh-2024-016342)

Supplemental Figure S4: The interrelationship diagraph developed during workshop 1

The diagraph was developed by the Jinja and Iganga groups during the plenary session as a precursor to the causal loop diagram.

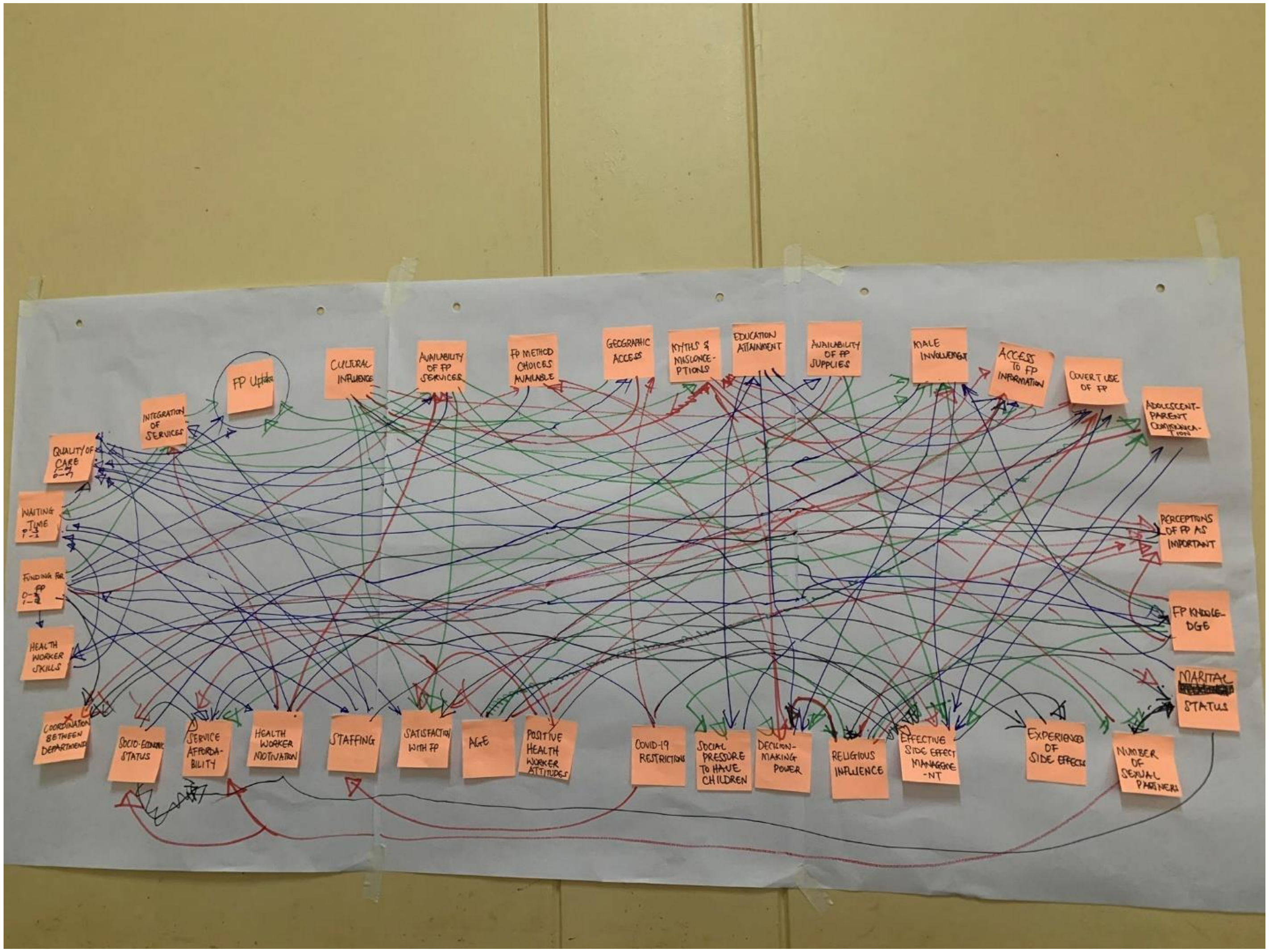

Supplement: online supplemental file 2 [file bmjgh-10-8-s002.pdf]
