# Supplementary material for: Using causal loop diagrams to examine the interrelationships between factors influencing family planning utilisation in urban east central Uganda
Source: BMJ Glob Health. 2025 Aug 17;10(8):e016342. doi: 10.1136/bmjgh-2024-016342 (PMC12359470; doi:10.1136/bmjgh-2024-016342)
Supplement: online supplemental file 3 [file bmjgh-10-8-s003.pdf]

**Supplemental Figure S6: The causal loop diagram developed through the Group Model Building exercises.**

The diagram shows the interrelationships between the factors that influence family planning use in Jinja and Iganga. Variables and links in green indicate relationships that apply to women and not men.

During subsequent refinement processes by the Core Modelling and research teams, four variables were removed. Waiting time, initially considered as a standalone factor, was merged with quality of care due to their intrinsic relationship. Health worker training opportunities was deemed redundant as an intermediary variable, adding little information to the model while unnecessarily complicating it. Similarly, covert use of family planning was identified as an adaptive behaviour rather than a direct driver of family planning utilisation. Lastly, the inclusion of COVID-19 restrictions was deemed transient, as its effects were limited to a specific period, leading to its exclusion from the model. On the other hand, the variable, care-seeking for side effect management, was introduced as an intermediate step between user reports of side effects and subsequent side effect management actions.

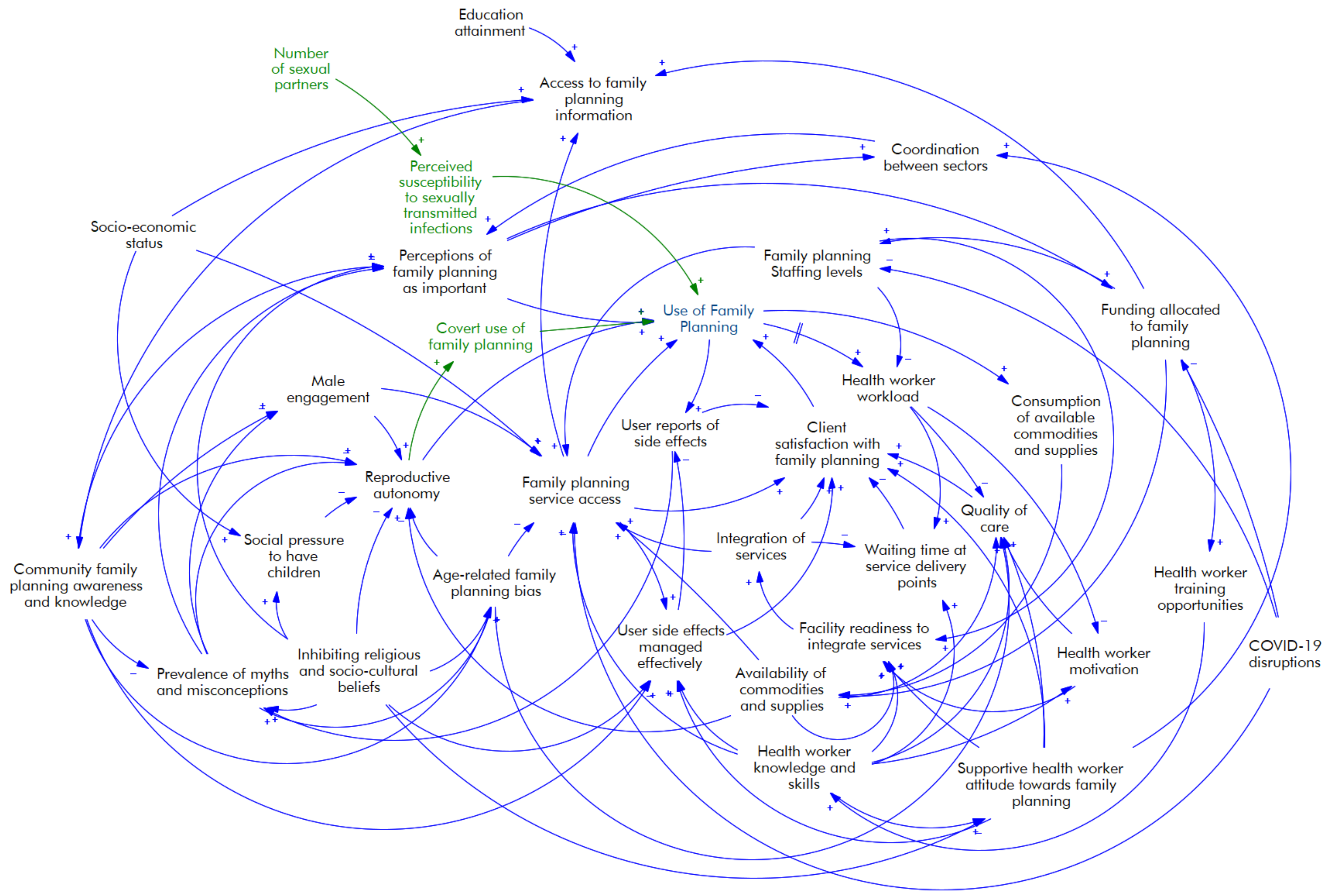
Education attainment
Number of sexual partners
Access to family planning information
Perceived susceptibility to sexually transmitted infections
Coordination between sectors
Socio-economic status
Perceptions of family planning as important
Family planning Staffing levels
Use of Family Planning
Covert use of family planning
Funding allocated to family planning
Male engagement
Health worker workload
Consumption of available commodities and supplies
User reports of side effects
Client satisfaction with family planning
Reproductive autonomy
Family planning service access
Quality of care
Community family planning awareness and knowledge
Social pressure to have children
Age-related family planning bias
Integration of services
Waiting time at service delivery points
Health worker training opportunities
Facility readiness to integrate services
User side effects managed effectively
Inhibiting religious and socio-cultural beliefs
Prevalence of myths and misconceptions
Availability of commodities and supplies
Health worker motivation
COVID-19 disruptions
Health worker knowledge and skills
Supportive health worker attitude towards family planning
