# Supplementary material for: Using causal loop diagrams to examine the interrelationships between factors influencing family planning utilisation in urban east central Uganda
Source: BMJ Glob Health. 2025 Aug 17;10(8):e016342. doi: 10.1136/bmjgh-2024-016342 (PMC12359470; doi:10.1136/bmjgh-2024-016342)

Supplemental Figure S5: The raw causal loop diagram during Workshop 2

The causal loop diagram was created during the second workshop to illustrate the interconnections between the factors influencing family planning utilisation in both Jinja and Iganga.

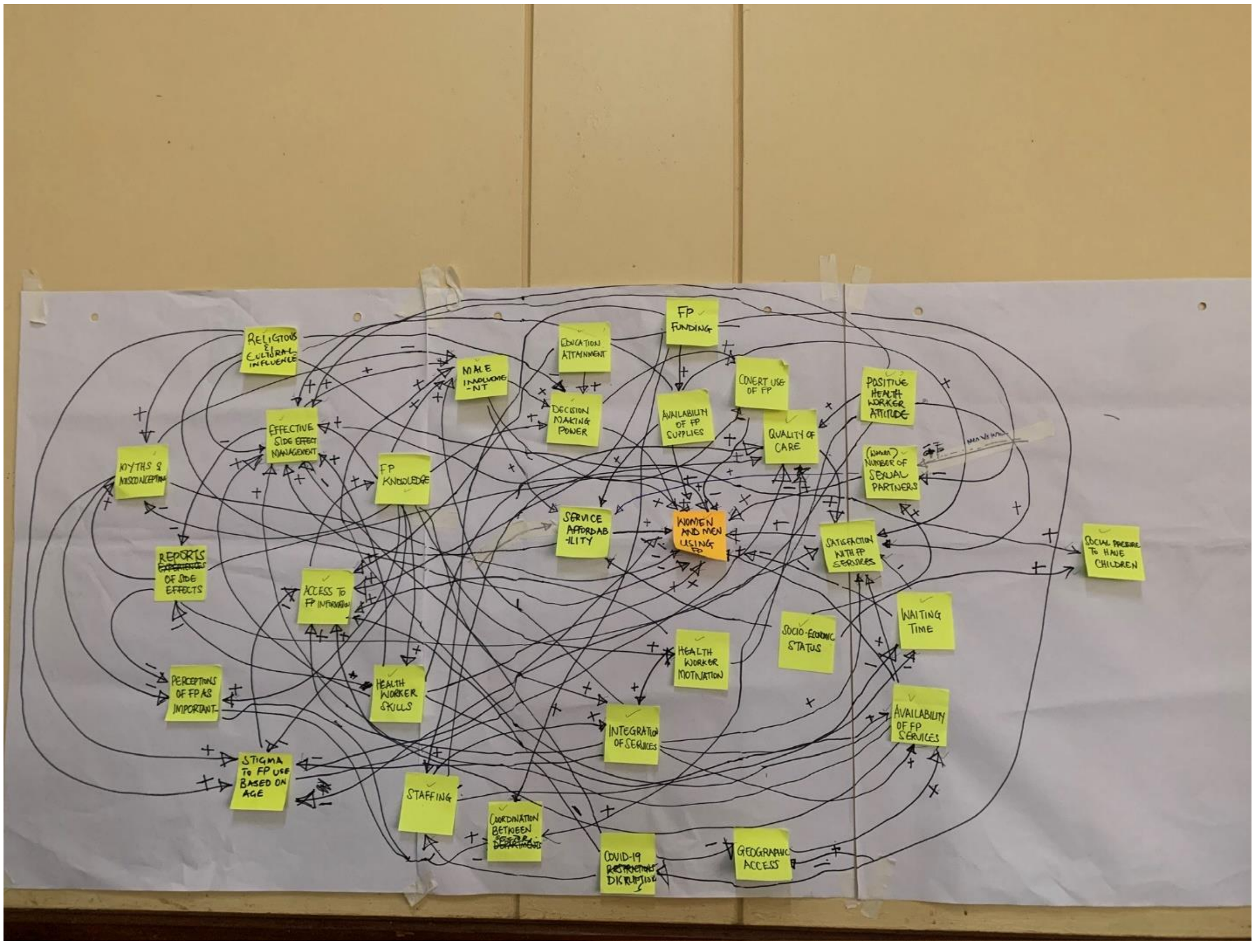

Supplement: online supplemental file 4 [file bmjgh-10-8-s004.pdf]
