# Supplementary material for: Using causal loop diagrams to examine the interrelationships between factors influencing family planning utilisation in urban east central Uganda
Source: BMJ Glob Health. 2025 Aug 17;10(8):e016342. doi: 10.1136/bmjgh-2024-016342 (PMC12359470; doi:10.1136/bmjgh-2024-016342)
Supplement: online supplemental file 5 [file bmjgh-10-8-s005.pdf]

Supplemental Table S1: Reflexivity statement

Criteria	Question	Response
Engagement	Has the research team engaged constructively with the reflexivity statement?	Yes, the research team has actively engaged with the reflexivity statement, critically reflecting on their positionality, biases, and power dynamics throughout the research process.
Co-development	Have the research partners co-developed the research study?	All research partners collaborated extensively in co-developing the research study, contributing to the study design, implementation, and interpretation of findings.
	Does the study address priority research questions for the LMIC partner(s)?	The study addresses family planning non-use, a critical and prioritised issue for LMICs such as Uganda, where unmet need for family planning remains a significant challenge.
Authorship	Is there a LMIC partner who is the first or last author?	The first author is a researcher from Uganda, reflecting the leadership role of LMIC partners in the study.
	How have LMIC early career researchers been incorporated as authors?	The first author, an early career researcher from Uganda, was given substantial responsibility and mentorship, promoting capacity building and professional growth.
Dissemination	How are data shared with LMIC partners to address research needs?	Data collection and management were led by Ugandan partners. The datasets are available for use by LMIC collaborators to explore additional research questions and policy needs.
	Is there open access funding to improve publication dissemination?	The paper is submitted for open access publication to increase access for all partners and communities, especially those in Uganda.
