# Supplementary material for: Using causal loop diagrams to examine the interrelationships between factors influencing family planning utilisation in urban east central Uganda
Source: BMJ Glob Health. 2025 Aug 17;10(8):e016342. doi: 10.1136/bmjgh-2024-016342 (PMC12359470; doi:10.1136/bmjgh-2024-016342)
Supplement: online supplemental file 6 [file bmjgh-10-8-s006.pdf]

Supplemental Table S2: Focus group discussion and interview transcripts analysed

Data Collection Method	Participant Groups	Total Number Analysed
Focus Group Discussions	Men	2 (1 from island community, 1 from mainland)
	Women	3 (1 from island community, 1 from mainland slum, 1 from mainland non-slum community)
	Adolescent boys	1 (mainland non-slum community)
	Adolescent girls	2 (1 from mainland slum, 1 from mainland non-slum community)
Key Informant Interviews	Health workers	4
	Urban health authorities	2
	Regional commodity manager	1
	FP partner organisation manager	1
In-Depth Interviews	Current female user	1
	Former female user	1
	Man opposed to FP	1
	Commercial sex worker	1

The data analysed included transcripts from eight Focus Group Discussions (FGDs) conducted with diverse groups: men (2), women (3), adolescent boys (1), and adolescent girls (2). These groups represented island communities, mainland slum areas, and mainland non-slum areas. Additionally, transcripts from eight Key Informant Interviews (KIIs) were analysed, featuring health workers (4), urban health authorities (2), a regional commodity manager (1), and a manager from a family planning (FP) partner organisation (1). Lastly, four In-Depth Interviews (IDIs) were analysed, capturing perspectives from a current female FP user, a former female FP user, a man opposed to FP, and a commercial sex worker.
